# Effects of arginine replacement with *L-*citrulline on the arginine/nitric oxide metabolism in chickens: An animal model without urea cycle

**DOI:** 10.1186/s40104-022-00817-w

**Published:** 2023-02-01

**Authors:** Victoria Anthony Uyanga, Lijing Sun, Yu Liu, Meiming Zhang, Jingpeng Zhao, Xiaojuan Wang, Hongchao Jiao, Okanlawon M. Onagbesan, Hai Lin

**Affiliations:** 1 Department of Animal Science, College of Animal Science and Veterinary Medicine, Shandong Provincial Key Laboratory of Animal Biotechnology and Disease Control, Shandong Agricultural University, No. 61 Daizong Street, Tai’an City, Shandong Province, 271018 China; 2 Department of Animal Physiology, Federal University of Agriculture, Ogun State, Abeokuta P.M.B, 2240 Nigeria

**Keywords:** Amino acids, Arginine, Broiler chicken, Citrulline, Intestinal health, Nutrient transporters, Tight junctions

## Abstract

**Background:**

This study examined the efficacy of *L-*citrulline supplementation on the arginine/nitric oxide metabolism, and intestinal functions of broilers during arginine deficiency. A total of 288 day-old Arbor Acre broilers were randomly assigned to either an arginine deficient basal diet (NC diet), NC diet + 0.50% *L-*arginine (PC diet), or NC diet + 0.50% *L-*citrulline (NCL diet). Production performance was recorded, and at 21 days old, chickens were euthanized for tissue collection.

**Results:**

The dietary treatments did not affect the growth performance of broilers (*P* > 0.05), although NC diet increased the plasma alanine aminotransferase, urate, and several amino acids, except arginine (*P* < 0.05). In contrast, NCL diet elevated the arginine and ornithine concentration higher than NC diet, and it increased the plasma citrulline greater than the PC diet (*P* < 0.05). The nitric oxide concentration in the kidney and liver tissues, along with the plasma and liver eNOS activities were promoted by NCL diet higher than PC diet (*P* < 0.05). In the liver, the activities of arginase 1, ASS, and ASL, as well as, the gene expression of *iNOS* and *OTC* were induced by PC diet greater than NC diet (*P* < 0.05). In the kidney, the arginase 1, ASS and ASL enzymes were also increased by PC diet significantly higher than the NC and NCL diets. Comparatively, the kidney had higher abundance of *nNOS*, *ASS*, *ARG2,* and *OTC* genes than the liver tissue (*P* < 0.05). In addition, NCL diet upregulated (*P* < 0.05) the mRNA expression of intestinal nutrient transporters (*EAAT3* and *PEPT1*), tight junction proteins (Claudin 1 and Occludin), and intestinal mucosal defense (*MUC2* and *pIgR*). The intestinal morphology revealed that both PC and NCL diets improved (*P* < 0.05) the ileal VH/CD ratio and the jejunal VH and VH/CD ratio compared to the NC fed broilers.

**Conclusion:**

This study revealed that NCL diet supported arginine metabolism, nitric oxide synthesis, and promoted the intestinal function of broilers. Thus, *L-*citrulline may serve as a partial arginine replacement in broiler's diet without detrimental impacts on the performance, arginine metabolism and gut health of chickens.

## Introduction

In poultry nutrition, feeding strategies that would promote optimal growth performance, as well as a reduction in feed cost and environmental impact are highly desired. In recent decades, the formulation of reduced crude protein diets along with the utilization of alternative protein sources and the inclusion of non-bound amino acids (AA) have become prevalent. This has facilitated the utilization of commercially available crystalline AA to fortify dietary profiles, replace protein-rich ingredients and support poultry production in an economically feasible approach [1]. These attempts to lower dietary crude proteins have produced several advantages including minimized feed cost, enhanced nutrient utilization, and improved animal welfare [2]. Feeding broilers with reduced crude protein diets have the potential to decrease environmental pollution emanating from nitrogen and ammonia emission, improvement in litter quality and welfare of birds, and to a certain extent, it diminishes the dependence on soybean meal to meet the dietary requirements of birds [3]. More so, it was reported that decreasing dietary crude protein levels by about 3% did not impede the growth performance and meat quality of chickens [4]. However, a significant decrease in dietary crude protein adversely affects the performance of broilers and increases carcass lipid deposition, thus, discouraging the adoption of reduced crude protein diets [5]. Reduced crude protein diets are typically formulated with increased inclusion of feed grains, reduction in protein-rich feed ingredients, as well as the selected inclusion of non-bound AA to meet the dietary needs of poultry. However, this formulations often increases the dietary starch to protein ratio, which may cause deleterious consequences [3, 5]. Therefore, it is imminent to investigate the limitations of AA inclusion and to formulate effective strategies for dietary crude protein reduction.

Asides from their primary role in protein synthesis and accretion, AA also functions to regulate key signaling pathways, causing significant changes in the gene expression, protein turnover, and physiological responses of animals [6, 7]. Studies have shown that dietary supplementation with AA such as arginine, citrulline, glutamine, leucine, and proline can modulate the gene expression and enhance the growth of the small intestine, as well as the skeletal muscle [8]. Dietary *L-*arginine supplementation promotes enterocyte proliferation, maintains intestinal barrier functions, and ameliorates intestinal inflammation [9, 10]. Thus, along with the conventional essential and non-essential AA, certain functional AA can also act to regulate key metabolic pathways, thus promoting the growth, development, health, and survival of animals [7]. Therefore, supplementation with these AA necessitates continuous assessment to derive optimal inclusions since variabilities can easily arise based on the bird's strain, environmental conditions, dietary protein status, and production objectives [11].

In poultry nutrition, arginine is an essential AA and its supply is necessary to support protein synthesis, immunity, reproduction, and production performance of birds [12]. Arginine is the fifth limiting AA for broiler chickens, and poultry has high arginine requirements due to their insufficient endogenous arginine synthesis. Also, dietary arginine requirements need to be optimized for modern broiler strains since they undergo rapid growth rate and protein deposition, and they also exhibit arginine–lysine antagonism [1]. Arginine and citrulline are present as metabolic intermediates in the urea cycle, and through enzymatic reactions, citrulline is catalyzed to form arginine, thus serving as a precursor for endogenous arginine synthesis, and consequently nitric oxide (NO) production [13]. Studies have shown that the utilization of commercially available AA such as crystalline arginine and citrulline can promote arginine availability in poultry [14, 15]. Citrulline can be used as a precursor for de novo arginine synthesis in the kidney, and various cells, as well as to supply nitrogen for protein homeostasis in peripheral tissues [16]. Importantly, citrulline exhibits a highly specific metabolism, since it can bypass splanchnic extraction, as such, it is not efficiently absorbed in the liver and intestine [16], rather it is transported to the kidney and other extra-renal tissues where the conditions for arginine synthesis are favorable [17].

Based on the demonstrated evidence that citrulline supplementation increased arginine levels in poultry [15], we hypothesized that supplementation of *L-*citrulline to an arginine-deficient diet would promote arginine availability, and consequently the physiological responses of broiler chickens. Thus, this study investigated the effects of arginine replacement with *L-*citrulline on the growth performance, AA profile, NO metabolism, and intestinal functions of broiler chickens.

## Materials and methods

### Animals and experimental design

A total of 288 1-day-old Arbor Acre chicks were brooded in battery cage units with environmentally controlled systems, continuous lighting, and ad libitum supply of feed and water. The chicks were brooded at 32 ± 1 °C with 55%–60% relative humidity for the first 3 d, then the temperature was gradually reduced until 24 ± 1 °C with 55%–60% relative humidity at 21 days of age. Chicks were weighed and randomly distributed into 3 treatments, 8 replicates of 12 chickens each. An arginine deficient basal diet (NC) was designed to contain 20.5% CP, and 12.55 MJ/kg ME (Table 1). The basal diet was formulated to meet or exceed the NRC recommendation for broiler chicks, except for arginine [18]. Other dietary treatments were subsequently formulated by supplementing the basal diet with arginine as the positive control (PC: NC diet + 0.50% *L-*arginine) or *L-*citrulline (NCL: NC + 0.50% *L-*Cit) (Table 1). Experimental diets were designed as isonitrogenous using alanine to balance the exclusion of arginine and they were fed to birds from day old until 21 days of age. Bodyweight (BW), bodyweight gain (BWG), feed intake (FI), and feed conversion ratio (FCR) were calculated per replicate of chickens.

**Table 1:** Composition and nutrient levels of experimental diets for 1–21 d (as-fed basis)

Ingredients, %	Dietary treatments		
	PC	NC	NCL
Corn (8.5% CP)	67.83	67.83	67.83
Soybean meal (43% CP)	16.86	16.86	16.86
Corn gluten meal (60% CP)	8.00	8.00	8.00
Wheat bran	0.77	0.77	0.77
Filler	0.53	-	0.27
Limestone	1.23	1.23	1.23
CaHPO_4_	1.88	1.88	1.88
NaCl	0.30	0.30	0.30
*L-*Lys·HCl (99%)	0.54	0.54	0.54
*DL-*Methionine (99%)	0.17	0.17	0.17
*L-*Threonine (99%)	0.11	0.11	0.11
*L-*Arginine (99%)	0.50	-	-
*L-*Citrulline (99%)	-	-	0.50
*L-*Alanine (99%)	0.78	1.80	1.04
Choline chloride (50%)	0.26	0.26	0.26
Vitamin premix	0.05	0.05	0.05
Mineral premix	0.20	0.20	0.20
Calculated nutrient levels			
Crude protein, %	20.5	20.5	20.5
Metabolizable energy, MJ/kg	12.55	12.55	12.55
Analyzed nutrient levels, %			
Crude protein	20.77	20.70	20.82
Dry matter	90.77	91.15	91.50
Crude fat	4.71	4.35	4.18
Ash	5.20	6.06	5.47
Calcium	1.03	1.23	1.09
Total phosphorus	0.66	0.68	0.69
Aspartate	1.06	1.03	1.08
Threonine	0.80	0.74	0.75
Serine	0.70	0.70	0.74
Glutamine	2.38	2.38	2.50
Glycine	0.47	0.45	0.47
Alanine	1.33	1.96	1.46
Cysteine	0.12	0.12	0.13
Valine	0.59	0.59	0.61
Methionine	0.19	0.17	0.22
Isoleucine	0.53	0.52	0.54
Leucine	1.42	1.44	1.50
Tyrosine	0.38	0.36	0.41
Phenylalanine	0.73	0.72	0.76
Lysine	0.95	0.93	0.94
Histidine	0.38	0.37	0.39
Arginine	1.21	0.68	0.73
Proline	0.86	0.86	0.90

*PC* Positive control, *NC* Negative control, *NCL* Negative control + *L-*citrulline

### Blood and tissue collection

At 21 days of age, one chicken per replicate was selected for sampling. About 3 mL of blood sample was collected into anti-coagulated tubes and later centrifuged at 4 °C, 1500 × *g* for 15 min to obtain the plasma. Plasma samples were stored at − 20 °C until analysis. Broilers were sacrificed via exsanguination and tissue samples were isolated, then snap-frozen in liquid nitrogen. Intestinal sections from the duodenum, jejunum, and ileum were collected as described by Chen et al. [19] and rinsed in saline. A portion of the ileum was opened to scrape the mucosa using glass slides into tubes. Samples collected were snap-frozen in liquid nitrogen and stored at − 80 °C until analysis. The weight of isolated organs was expressed as a percentage of the final body weight of chickens to obtain the relative organ index.

### Determination of plasma biochemistry

About 500 µL plasma was used for the determination of metabolites including alanine aminotransferase (ALT), aspartate aminotransferase (AST), urea, urate, creatine kinase (CK), glucose (GLU), triglyceride (TG), total cholesterol (TCHO) and lactate dehydrogenase (LDH). The plasma biochemistry was determined automatically using the Hitachi L*-*7020 automatic biochemical analyzer (Hitachi High-Technologies Corp., Tokyo, Japan).

### Determination of analyzed feed nutrients and plasma AA profile

Feed samples were analyzed for dry matter, crude fat, crude fiber, and ash using standardized methods [20]. For AA analysis in experimental diets, feed samples were hydrolyzed with 6 mol/L HCl (Yantai Yuandong Fine Chemical Industry, Shandong, China) at 110 °C for 22 h. After hydrolysis, samples were analyzed by ion exchange chromatography with postcolumn ninhydrin detection using Hitachi L-8900 AA Analyzer (Hitachi High-Technologies Crop., Tokyo, Japan), as previously described [21].

For plasma AA determination, 40 mg salicylic acid (Tianjin Haitong Chemical Industrial Co., Ltd., Tianjin, China) was added to 800 µL plasma for deproteinization [15]. The samples were vortexed and stored at 4 °C overnight. Afterward, the samples were centrifuged at 4 °C, 12,000 r/min for 30 min, and the supernatant was collected. Supernatants were filtered (0.22 μm) into well*-*labeled tubes and about 500 µL of the filtrate was used for detection of AA contents via ion-exchange chromatography with the Hitachi L*-*8900 AA Analyzer.

### Determination of nitric oxide concentration and total nitric oxide synthase activity

Nitric oxide (NO) and total nitric oxide synthase (tNOS) were determined using commercial test kits (Jiancheng Bioengineering Institute, Jiangsu, China) according to the manufacturer’s instructions. The reaction absorbance for NO concentration was determined at 550 nm using a microplate reader (Elx808, Bio-Tek, Winooski, Vermont, USA), while tNOS was read at 530 nm using a spectrophotometer (Beijing PGeneral, China).

### Determination of enzymes associated with arginine metabolism

Enzymes involved in arginine metabolism including endothelial nitric oxide synthase (eNOS), arginase 1, argininosuccinate synthetase (ASS), and argininosuccinate lyase (ASL) were determined using chicken ELISA kits (Shanghai MLBIO Biotechnology Co., Ltd., China). The assay was measured at 450 nm using a microplate reader (Elx808) and the standard curve was used to compute the sample concentration.

### Real-time polymerase chain reaction

The total RNA from the kidney and liver tissues were extracted using the NcmZol reagent (NCM Biotech, Shanghai, China), and the RNA concentration and purity were determined using the DS-11 spectrophotometer (Denovix Incorporated, Delaware, United States). Reverse transcription was carried out using the HiFiScript cDNA synthesis kit (CWBIO, Beijing, China) in a 20-μL final reaction volume and the cDNA synthesis reaction was carried out using the Genemate T960 Touch thermocycler (Heal Force Bio-Meditech Holdings Limited, Shanghai, China). The cDNA target sequence was quantified using MagicSYBR mixture (CWBIO, Beijing, China), in a 20-μL volume with appropriate primers (Table 2). The real*-*time RT-qPCR was performed using ABI QuantStudio5 Real*-*Time PCR Instrument (Applied Biosystems, ThermoFisher Scientific, Massachusetts, United States). The primers were normalized against β-actin as the housekeeping gene, while the positive control (PC) diet was used as the calibrator. The relative expression of the target genes were analyzed using the 2^−ΔΔCT^ method.

**Table 2:** List of primers used for real*-*time PCR analysis

Target gene	Primer direction	Primer sequence (5′→3′)	Genebank accession No.
β-Actin	Forward	TGCGTGACATCAAGGAGAAG	NM_001145490.1
	Reverse	TGCCAGGGTACATTGTGGTA	
*b*^*0,*+^*AT*	Forward	TGTGTTGCTCTCTAACTGGCTG	NM_001199133.1
	Reverse	CCTCCTTTCTGTTGTCCTGTTC	
*EAAT3*	Forward	ACCCTTTTGCCTTGGAAACT	XM_424930
	Reverse	TTGAGATGTTTGCGTGAAG	
*PepT1*	Forward	ACACGTTTGTTGCTCTGTGC	NM_204365
	Reverse	GACTGCCTGCCCAATTGTAT	
Claudin1	Forward	ATGACCAGGTGAAGAAGATGC	NM_001013611.2
	Reverse	TGCCCAGCCAATGAAGAG	
Occludin	Forward	GGTTCCTCATCGTCATCCTGCTC	XM_025144248.1
	Reverse	GCCTCGTTCTTCACCCACTCCT	
*ZO-1*	Forward	CTTCAGGTGTTTCTCTTCCTCCTC	XM_015278981.2
	Reverse	CTGTGGTTTCATGGCTGGATC	
*pIgR*	Forward	GGATCTGGAAGCCAGCAAT	ENSGALT00000001353
	Reverse	GAGCCAGAGCTTTGCTCAGA	
*MUC2*	Forward	CCCTGGAAGTAGAGGTGACTG	XM_001234581.3
	Reverse	TGACAAGCCATTGAAGGACA	
*nNOS*	Forward	CTCGGATGCACGGAAGTCCT	XM_004934480.1
	Reverse	CGTGAACCCAGCCCAAACAC	
*iNOS*	Forward	GTGGTATGCTCTGCCTGCTGTTG	NM_204961.1
	Reverse	GTCTCGCACTCCAATCTCTGTTCC	
*eNOS*	Forward	GGATGTGCTGCACGGTCTGC	JQ434761.1
	Reverse	AGGACGTGCTGCGGACACAG	
*ASS*	Forward	GACACCTCCTGCATTCTGGT	NM_001013395
	Reverse	CTTCTGGGCTGCATCAAAGT	
*ARG2*	Forward	GCCAACTGTACGACTTTGGAG	AB159222
	Reverse	AGCTGTGTCCAGCAGCTACC	
*CPS1*	Forward	GCCAACAGAGGACAGAACCA	NM_001045841
	Reverse	CAGGTGGGAGGGTAGAACTG	
*OTC*	Forward	ACCTCCACTCCCTTGTCCTC	NM_204910
	Reverse	TGTGCAGCTCCTTGTAATGC	

*b*^*0,+*^*AT*:b^0,+^ AA transporter; *EAAT3*: Excitatory AA transporter 3; *PepT1*: Peptide transporter 1;* ZO-1*: Zona Occludens 1; *MUC2*: Mucin 2; *pIgR*: Polymeric Ig receptor; *nNOS*: Neuronal nitric oxide synthase; *iNOS*: Inducible nitric oxide synthase; *eNOS*: Endothelial nitric oxide synthase; *ASS*: Argininosuccinate synthetase; *ARG2*: Arginase 2; *CPS1*: Carbamoyl phosphate synthetase 1; *OTC*: Ornithine transcarbamylase

### Intestinal histology 

About 5 cm section of ileum, duodenum, and jejunum were fixed in 4% paraformaldehyde (Wuhan Servicebio Technology Co., Ltd., Wuhan, China). The intestinal segments were trimmed, processed, and embedded in paraffin wax. 5 µm section of each sample was placed on a glass slide and stained with hematoxylin and eosin for morphometric examination. Slides were visualized using Olympus CX-41 phase contrast microscope (CK-40, Olympus, Tokyo, Japan). The distance between the top of the villus to the villus-crypt junction was measured as villus height (VH), while the distance from the villus-crypt junction down to the bottom of the crypt was measured as crypt depth (CD) [10, 19]. Three measurements were taken per slide and the average was obtained for analysis. The VH to CD ratio was computed per observation.

### Statistical analysis

Data collected were analyzed with a one-way analysis of variance using the GLM procedure of SAS (SAS version 8.1; SAS Institute Inc., Cary, North Carolina, USA). The gene expression for arginine metabolizing enzymes was analyzed using two-way ANOVA to evaluate the main effects of tissue (kidney vs. liver), diet (PC vs. NC vs. NCL), and their interaction. Means separation was performed using Duncan’s Multiple Range Test and treatment effects were considered statistically significant at a probability of *P* < 0.05.

## Results

### Production performance and relative organ weights of broilers

The production performance of broilers did not differ among the dietary treatments (Table 3). As such, the BW, BWG, FI, and FCR of broilers fed with either the PC, NC or NCL diets did not vary significantly (*P* > 0.05). Similarly, the relative organ index of the liver, kidney, spleen, bursa, thymus, ileum, duodenum, and breast muscle of broilers were not affected by dietary treatments as given in Table 4 (*P* > 0.05). However, the relative weight of the jejunum was lowered by the PC diet compared to the NC and NCL diets (*P* < 0.05). In addition, the thigh muscle was reduced by the NC diet compared to the PC and NCL diets (*P* < 0.05).

**Table 3:** Production performance and relative organ weights of broilers fed arginine deficient diets supplemented with arginine or *L-*citrulline

	Dietary treatments			*P* value
	PC	NC	NCL	
BW, g/bird				
0 d	45.69 ± 0.34	46.04 ± 0.04	46.03 ± 0.03	0.370
14 d	279.73 ± 7.67	292.31 ± 8.23	275.09 ± 6.06	0.256
21 d	511.75 ± 6.49	511.25 ± 14.23	496.50 ± 8.42	0.501
BWG, g/bird				
0–13 d	234.05 ± 7.38	246.27 ± 8.23	229.06 ± 6.08	0.251
14–21 d	232.02 ± 11.57	218.94 ± 15.40	221.41 ± 9.93	0.738
0–21 d	466.06 ± 6.66	465.21 ± 14.21	450.47 ± 8.41	0.495
FI, g/bird				
0–13 d	368.72 ± 20.31	387.78 ± 7.47	367.14 ± 34.80	0.792
14–21 d	317.38 ± 5.96	317.88 ± 16.61	300.38 ± 14.63	0.576
0–21 d	686.10 ± 21.29	705.66 ± 21.54	667.52 ± 37.04	0.627
FCR	1.47 ± 0.05	1.52 ± 0.04	1.48 ± 0.09	0.864

BW, Body weight; BWG, Body weight gain; FCR, Feed conversion rate; PC: Positive control; NC: Negative control; NCL: Negative control + *L-*citrulline. Data are presented as mean ± SEM (*n* = 8)

**Table 4:** Relative organ index of broilers fed arginine deficient diets supplemented with arginine or *L-*citrulline

Parameters, %	Dietary treatments			*P* value
	PC	NC	NCL	
Liver	2.36 ± 0.07	2.51 ± 0.11	2.49 ± 0.11	0.486
Kidney	0.59 ± 0.02	0.59 ± 0.02	0.60 ± 0.03	0.914
Spleen	0.08 ± 0.01	0.10 ± 0.01	0.10 ± 0.01	0.339
Bursa	0.21 ± 0.02	0.21 ± 0.02	0.20 ± 0.03	0.942
Thymus	0.22 ± 0.01	0.24 ± 0.02	0.19 ± 0.02	0.087
Ileum	1.07 ± 0.03	1.10 ± 0.07	1.20 ± 0.4	0.190
Jejunum	1.43 ± 0.05^b^	1.62 ± 0.07^a^	1.69 ± 0.03^a^	0.008
Duodenum	0.99 ± 0.05	1.06 ± 0.07	1.04 ± 0.04	0.682
Breast muscle	11.40 ± 0.64	10.70 ± 0.35	10.63 ± 0.28	0.423
Thigh muscle	12.79 ± 0.52^a^	11.07 ± 0.41^b^	12.76 ± 0.45^a^	0.029

*PC:* Positive control, *NC:* Negative control, *NCL*: Negative control + *L-*citrulline. Data are presented as mean ± SEM (*n* = 8). ^a,b^Means with different superscript letters are significantly different (*P* < 0.05) within the same row

### Plasma biochemistry and AA profile of broilers

Plasma metabolites such as urea, CK, GLU, TG, TCHO, and LDH were not significantly influenced (*P* > 0.05) by the dietary treatments (Table 5). The ALT concentration was decreased (*P* < 0.05) in broilers fed NCL diet compared to the PC and NC diets which were similar. In addition, plasma AST was decreased (*P* < 0.05) in both the NC and NCL diet groups compared to the PC diet. The urate content was higher in NC fed birds than with the PC and NCL diets (*P* < 0.05).

**Table 5:** Plasma biochemistry of broilers fed arginine deficient diets supplemented with arginine or *L-*citrulline

Parameters	Dietary treatments			*P* value
	PC	NC	NCL	
ALT, U/L	5.71 ± 0.42^a^	5.88 ± 0.55^a^	4.00 ± 0.57^b^	0.034
AST, U/L	204.88 ± 7.18^a^	183.88 ± 4.11^b^	181.88 ± 7.98^b^	0.043
Urea, µmol/L	0.95 ± 0.05	0.87 ± 0.03	0.91 ± 0.03	0.386
Urate, µmol/L	370.38 ± 42.62^b^	633.29 ± 74.65^a^	446.88 ± 60.71^b^	0.018
CK, U/L	2374.88 ± 408.74	1608.88 ± 159.98	1852.75 ± 278.86	0.207
GLU, mmol/L	12.78 ± 0.28	12.57 ± 0.43	12.73 ± 0.22	0.892
TG, mmol/L	0.23 ± 0.03	0.23 ± 0.03	0.25 ± 0.02	0.843
TCHO, mmol/L	2.95 ± 0.16	2.63 ± 0.11	2.76 ± 0.10	0.226
LDH, U/L	661.38 ± 35.84	589.75 ± 37.62	597.88 ± 34.22	0.323

ALT: Alanine aminotransferase; AST: Aspartate aminotransferase, CK: Creatine kinase; GLU: Glucose; TG: Triglyceride; TCHO: Total cholesterol; LDH: Lactate dehydrogenase; PC: Positive control; NC: Negative control; NCL: Negative control + *L-*citrulline. Data are presented as mean ± SEM (*n* = 8). ^a,b^Means with different superscript letters are significantly different (*P* < 0.05) within the same row

Among the essential AA shown in Table 6, the plasma concentrations of threonine, leucine, valine, and cysteine were increased by the NC diet significantly higher than both the PC and NCL diets (*P* < 0.05). However, the leucine content was significantly decreased with the PC diet compared to the NCL diet (*P* < 0.05). Importantly, the arginine concentration was lowered by the NC diet compared to both the PC and NCL diets (*P* < 0.05).

**Table 6:** Plasma AA profile of broilers fed arginine deficient diets supplemented with arginine or *L-*citrulline

AA, ng/µL	Dietary treatments			*P* value
	PC	NC	NCL	
Essential AA				
Threonine	72.48 ± 4.58^b^	88.59 ± 6.08^a^	62.19 ± 4.37^b^	0.005
Methionine	9.85 ± 0.46	12.00 ± 0.93	11.14 ± 0.67	0.125
Lysine	39.33 ± 8.93	24.75 ± 4.57	52.04 ± 10.29	0.090
Histidine	13.01 ± 1.38	15.28 ± 1.76	10.48 ± 1.13	0.086
Phenylalanine	25.55 ± 1.09	30.41 ± 0.84	28.93 ± 1.96	0.059
Arginine	60.89 ± 4.14^a^	24.49 ± 1.62^b^	51.00 ± 5.43^a^	< .000
Isoleucine	10.86 ± 0.70	13.36 ± 0.77	12.25 ± 0.94	0.114
Leucine	26.97 ± 2.53^c^	39.15 ± 1.42^a^	32.75 ± 1.54^b^	0.001
Valine	20.19 ± 0.97^b^	27.39 ± 1.27^a^	20.73 ± 1.60^b^	0.001
Cysteine	14.51 ± 1.11^b^	22.14 ± 1.07^a^	16.70 ± 1.44^b^	0.001
Tryptophan	8.10 ± 0.54	6.52 ± 0.74	7.40 ± 0.76	0.285
Glycine	52.43 ± 3.26	57.05 ± 3.46	45.96 ± 3.46	0.091
Non-essential AA				
Citrulline	6.22 ± 0.71^b^	9.72 ± 0.85^a^	9.87 ± 0.99^a^	0.010
Ornithine	3.47 ± 0.18^ab^	2.68 ± 0.25^b^	3.76 ± 0.36^a^	0.030
Aspartate	15.43 ± 1.57^b^	34.25 ± 2.99^a^	21.12 ± 1.99^b^	< .000
Serine	58.91 ± 3.59^b^	75.76 ± 5.98^a^	59.82 ± 5.97^b^	0.062
Proline	47.61 ± 4.48^b^	71.42 ± 3.62^a^	49.10 ± 4.31^b^	0.001
Glutamate	36.29 ± 1.39	39.98 ± 2.62	37.10 ± 1.88	0.415
Alanine	87.94 ± 3.77^b^	134.47 ± 10.06^a^	88.39 ± 4.67^b^	< .000
Sarcosine	2.47 ± 0.18^ab^	2.84 ± 0.04^a^	2.15 ± 0.14^b^	0.006
Cystathionine	5.45 ± 0.42^b^	7.23 ± 0.42^a^	6.35 ± 0.42^ab^	0.024
Taurine	12.10 ± 0.91	13.44 ± 1.81	18.21 ± 4.77	0.335
Tyrosine	40.55 ± 2.34	45.80 ± 2.38	42.74 ± 4.61	0.535
Beta-Alanine	4.64 ± 0.36	4.05 ± 0.21	4.58 ± 0.21	0.255
Gamma-aminobutyric acid	0.96 ± 0.11	0.60 ± 0.09	0.85 ± 0.11	0.066
3Methylhistidine	2.15 ± 0.25	1.70 ± 0.20	1.71 ± 0.29	0.371
Anserine	7.00 ± 0.25	7.53 ± 0.56	8.06 ± 0.61	0.350
Carnosine	3.48 ± 0.37	3.41 ± 0.32	2.79 ± 0.14	0.211

*PC:* Positive control, *NC:* Negative control, *NCL:* Negative control + *L-*citrulline

Data are presented as mean ± SEM (*n* = 8). ^a,b,c^ Means with different superscript letters are significantly different (*P* < 0.05) within the same row

Among the non-essential AA and related peptides, the plasma concentrations of aspartate, serine, proline, and alanine were increased by the NC diet (*P* < 0.05), although they did not differ between the PC and NCL diet. The citrulline content was significantly increased in both the NC and NCL groups compared to the PC diet (*P* < 0.05). Likewise, NCL diet increased the ornithine levels higher than the NC diet. In contrast, NC diet increased the sarcosine and cystathionine contents higher than the NCL and PC diets, respectively (*P* < 0.05).

### Nitric oxide concentration

**Fig. 1:**
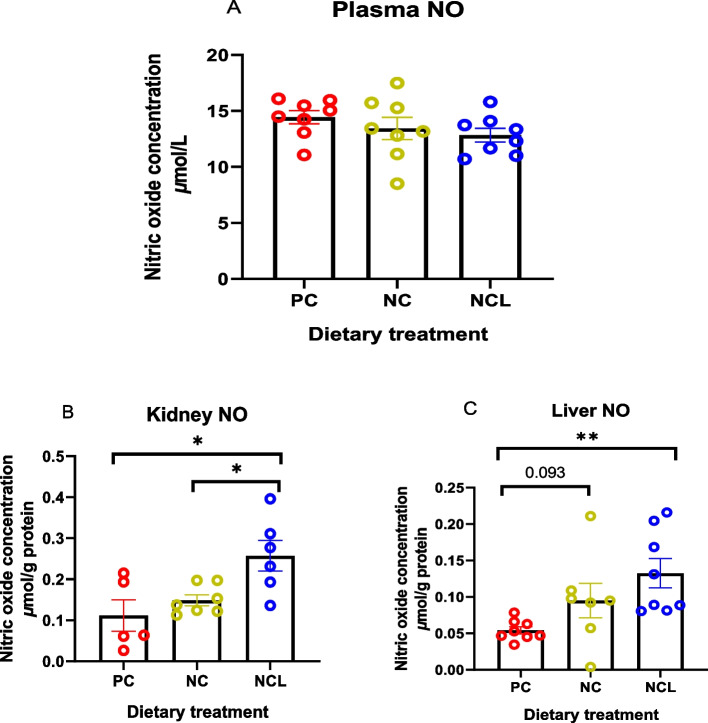
Nitric oxide (NO) concentration in broilers fed arginine deficient diets supplemented with arginine or *L-*citrulline. (**A**) Plasma NO (**B**) Kidney NO (**C**) Liver NO. PC: Positive control; NC: Negative control; NCL: Negative control + *L-*citrulline. Data are presented as mean ± SEM (*n* = 6 to 8). Data are significantly different at *P < 0.05; **P < 0.01

The plasma NO was not significantly affected (*P* > 0.05) by the dietary treatments of PC, NC, and NCL diets (Fig. 1A). However, broilers fed NCL diet exhibited increased kidney NO concentration compared to the broilers fed either NC or PC diets (Fig. 1B; *P* < 0.05). Also, the NO concentration in the liver was significantly increased by the NCL diet compared to the PC diet (*P* < 0.05), whereas, NC diet showed a tendency to increase the liver NO content more than the PC diet (Fig. 1C).

### Activities of arginine metabolizing enzymes

**Fig. 2:**
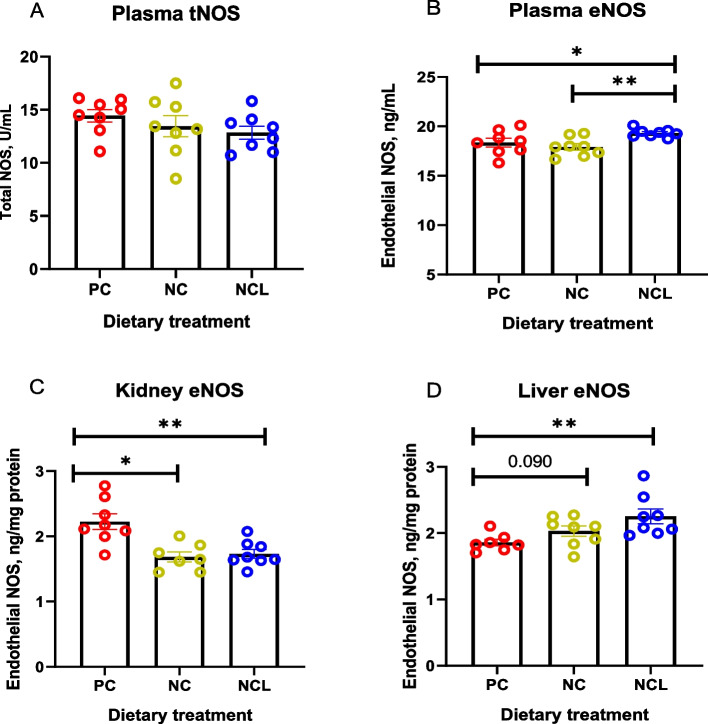
Enzyme activity of nitric oxide synthase isoforms in broilers fed arginine deficient diets supplemented with arginine or *L-*citrulline. (**A**) Plasma tNOS (**B**) Plasma eNOS (**C**) Kidney eNOS (**D**) Liver eNOS. PC: Positive control; NC: Negative control; NCL: Negative control + *L-*citrulline. Data are presented as mean ± SEM (*n* = 7 to 8). Data are significantly different at *P ≤ 0.05; **P < 0.01; ***P < 0.001

The enzyme activities for tNOS and eNOS are presented in Fig. 2. The plasma tNOS did not differ among dietary treatments (Fig. 2A; *P* > 0.05). However, the plasma eNOS activity was promoted with NCL diet higher than both the PC and NC diets (Fig. 2B; *P* ≤ 0.05). In contrast, the kidney eNOS activity was increased (*P* < 0.05) in the PC fed group compared to both the NC and NCL diets (Fig. 2C). Also, the liver eNOS activity was increased by NCL diet higher than the PC fed group (Fig. 2D; *P* < 0.05).

**Fig. 3:**
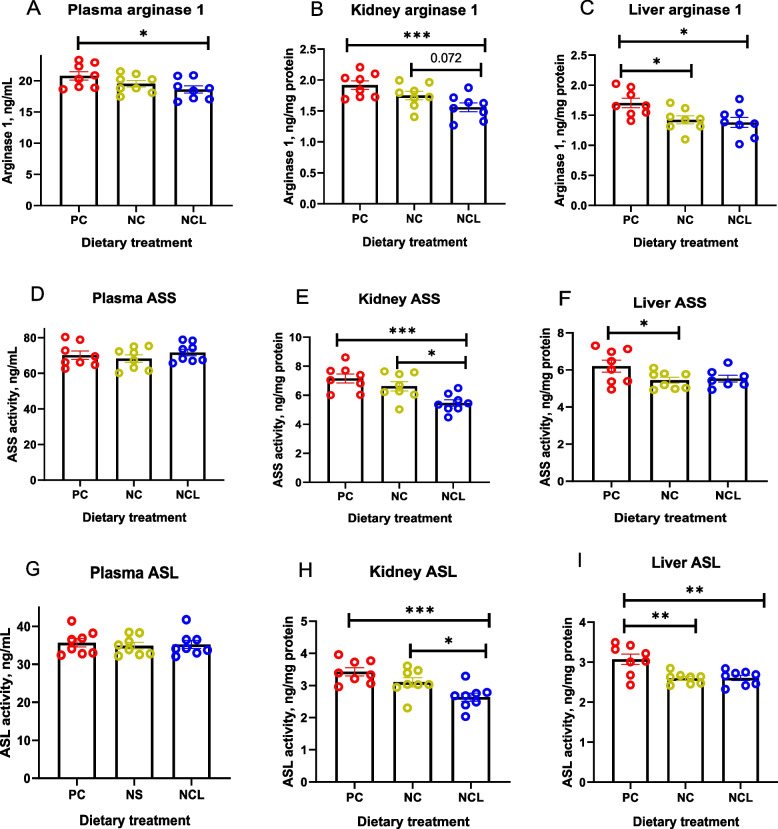
Activity of arginine metabolizing enzymes in broilers fed arginine deficient diets supplemented with arginine or *L-*citrulline. (**A**) Plasma arginase (**B**) Kidney arginase (**C**) Liver arginase (**D**) Plasma ASS (**E**) Kidney ASS (**F**) Liver ASS (**G**) Plasma ASL (**H**) Kidney ASL (**I**) Liver ASL. PC: Positive control; NC: Negative control; NCL: Negative control + *L-*citrulline. Data are presented as mean ± SEM (*n* = 8). Data are significantly different at *P ≤ 0.05; **P < 0.01; ***P < 0.001

The plasma arginase 1 activity was significantly increased with the PC diet than the NCL diet (Fig. 3A). Arginase 1 was also higher in the kidney of PC-fed broilers than with the NCL diet (*P* < 0.05). The NC diet also tended to increase the kidney arginase 1 higher than the NCL diet (Fig. 3B). In the liver, arginase 1 was promoted (*P* < 0.05) by the PC diet compared to the NC and NCL diets, which were similar (Fig. 3C). Figure 3D shows that the plasma ASS activity was unaffected (*P* > 0.05) by the dietary treatments, whereas, the kidney ASS activity was decreased (*P* < 0.05) by NCL diet compared to the PC and NC groups (Fig. 3E). In addition, the liver ASS activity was increased (*P* ≤ 0.05) by the PC diet, since it was relatively higher than the NC diet (Fig. 3F). Furthermore, the plasma ASL activity did not differ between the dietary groups (Fig. 3G), whereas the kidney ASL activity was decreased with NCL diet compared to PC and NC groups (Fig. 3H; *P* < 0.05). Similarly, ASL activity in the liver was decreased (*P* < 0.05) by both NC and NCL diets compared to the PC diet (Fig. 3I).

### Gene expression of arginine metabolizing enzymes

**Fig. 4:**
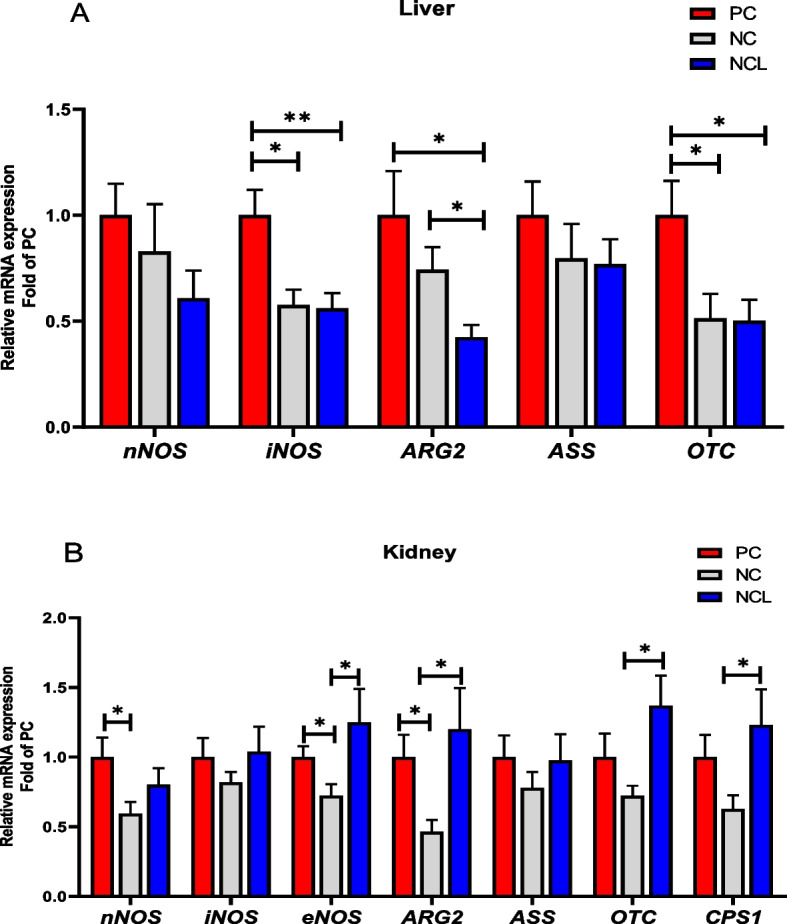
Gene expression of arginine metabolizing enzymes in the tissues of broilers fed arginine deficient diets supplemented with arginine or *L-*citrulline. (**A**) Liver (**B**) Kidney. PC: Positive control; NC: Negative control; NCL: Negative control + *L-*citrulline. Data are presented as mean ± SEM (*n* = 8). Data are significantly different at *P < 0.05; **P < 0.01

The relative mRNA expression of the various enzymes involved in arginine metabolism are shown in Fig. 4. In the liver (Fig. 4A), the *nNOS* and *ASS* expression were unaffected by dietary treatments (*P* > 0.05), whereas, the *iNOS*, *ARG2* and *OTC* expression were significantly upregulated by the PC diet compared to the NCL diet (*P* < 0.05). Also, PC diet increased the liver *iNOS* and *OTC* expression higher than the NC diet. In addition, *ARG2* expression was modulated by NC diet compared to the NCL group (*P* < 0.05). In the kidney (Fig. 4B), the *iNOS* and *ASS* expression did not differ among dietary treatments (*P* > 0.05). However, feeding with PC diet significantly upregulated the *nNOS*, *eNOS* and *ARG2* expression higher than the NC diet (*P* < 0.05). Furthermore, NCL diet also upregulated the *eNOS*, *ARG2, OTC,* and *CPS 1* expression higher than the NC diet (*P* < 0.05).

**Fig. 5:**
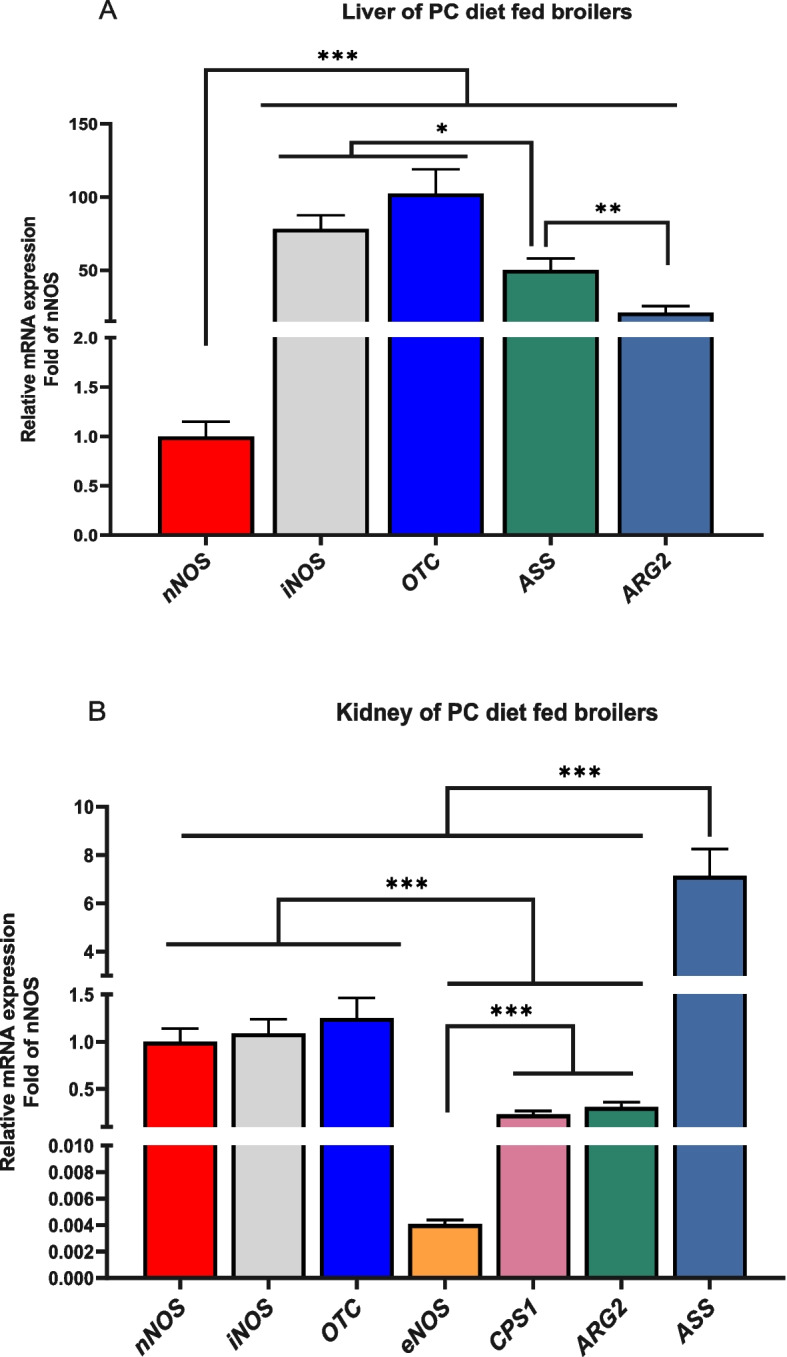
Tissue expression profile of arginine metabolizing enzymes in broilers fed arginine deficient diets supplemented with arginine (PC diet) (**A**) Liver (**B**) Kidney. Data are presented as mean ± SEM (*n* = 8). Data are significantly different at *P < 0.05; **P < 0.01; ***P < 0.001

**Fig. 6:**
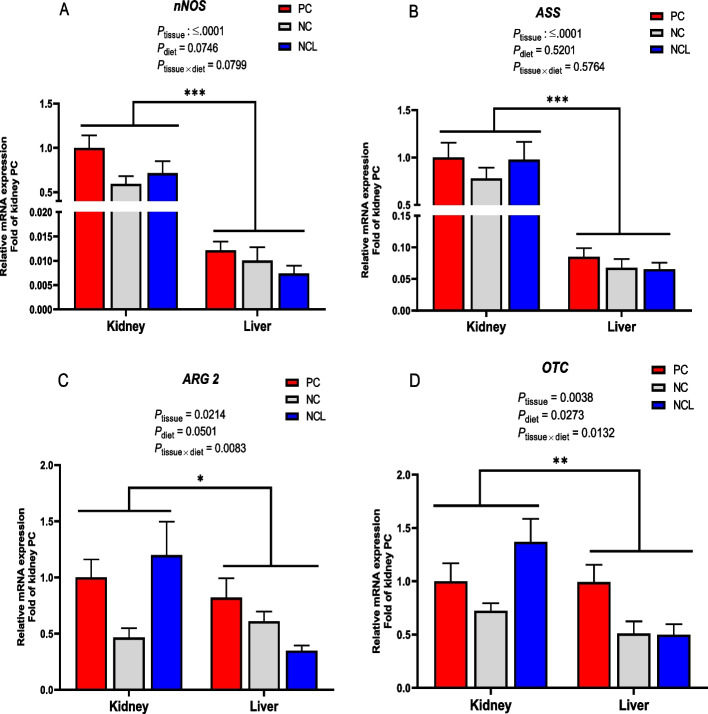
Comparison of the relative gene expression of arginine metabolizing enzymes in the kidney and liver tissues of broilers fed arginine deficient diets supplemented with arginine or *L-*citrulline. (**A**) nNOS (**B**) ASS (**C**) ARG2 (**D**) OTC. PC: Positive control; NC: Negative control; NCL: Negative control + *L-*citrulline. Data are presented as mean ± SEM (*n* = 8). Data are significantly different at *P < 0.05; **P < 0.01; ***P < 0.001

It was also observed that the relative abundance of arginine metabolizing genes differed (*P* < 0.05) among each other (Fig. 5). In the liver, *iNOS* and *OTC* genes had the highest expression, followed by *ASS*, then *ARG2* and the lowest was *nNOS* expression (Fig. 5A; *P* < 0.05). In the kidney, *ASS* was highly expressed, followed by *nNOS*, *iNOS*, and *OTC* which were similar, then *CPS1* and *ARG2,* while *eNOS* had the lowest gene expression (Fig. 5B; *P* < 0.05). A comparative assessment of the relative abundance of arginine metabolizing enzymes between the kidney and liver tissue is shown in Fig. 6. It was observed that the *nNOS, ASS, ARG2,* and *OTC* were highly abundant (*P*_*tissue*_ < 0.05) in the kidney compared to their expression in the liver (Fig. 6A–D).

### Gene expression of nutrient transporters in the intestine of broilers

**Fig. 7:**
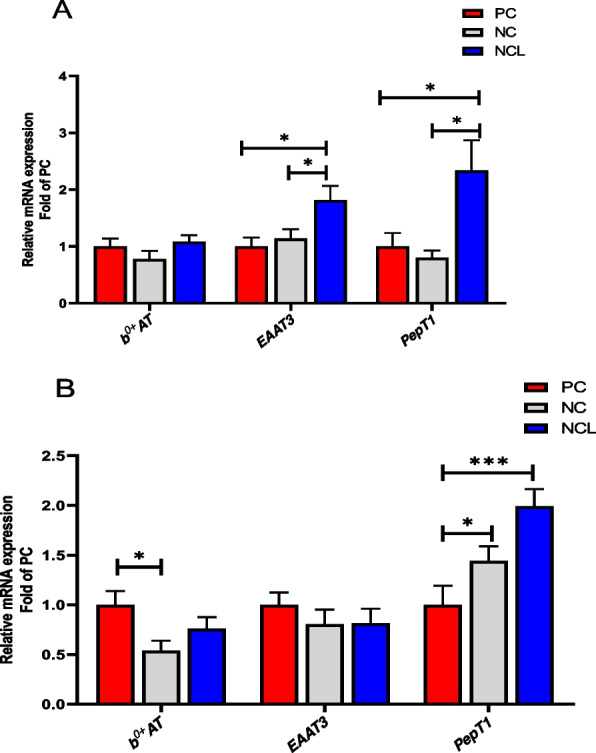
Gene expression of nutrient transporters in the intestine of broilers fed arginine deficient diets supplemented with arginine or *L-*citrulline. (**A**) Ileum tissue (**B**) Jejunum tissue. PC: Positive control; NC: Negative control; NCL: Negative control + *L-*citrulline. Data are presented as mean ± SEM (*n* = 8). Data are significantly different at *P < 0.05; ***P < 0.001

The ileal expression of *b*^0,+^*AT* transporter did not differ between the dietary treatments (Fig. 7A; *P* > 0.05), whereas, the *EAAT3* and *PepT1* transporters were similarly influenced (*P* < 0.05). Feeding with NCL diet upregulated the mRNA expression of *EAAT3* and *PepT1* in the ileum of broilers compared to the PC and NC diets (*P* < 0.05).

In the jejunum, the mRNA expression of *EAAT3* was unchanged (*P* > 0.05), whereas, PC diet upregulated *b*^0,+^*AT* expression higher than the NC diet (Fig. 7B). In addition, feeding with NC and NCL diets significantly upregulated *PepT1* mRNA expression in the jejunum compared to the PC diet (Fig. 7B; *P* < 0.05).

### Gene expression of tight junction proteins in the intestine of broilers

**Fig. 8:**
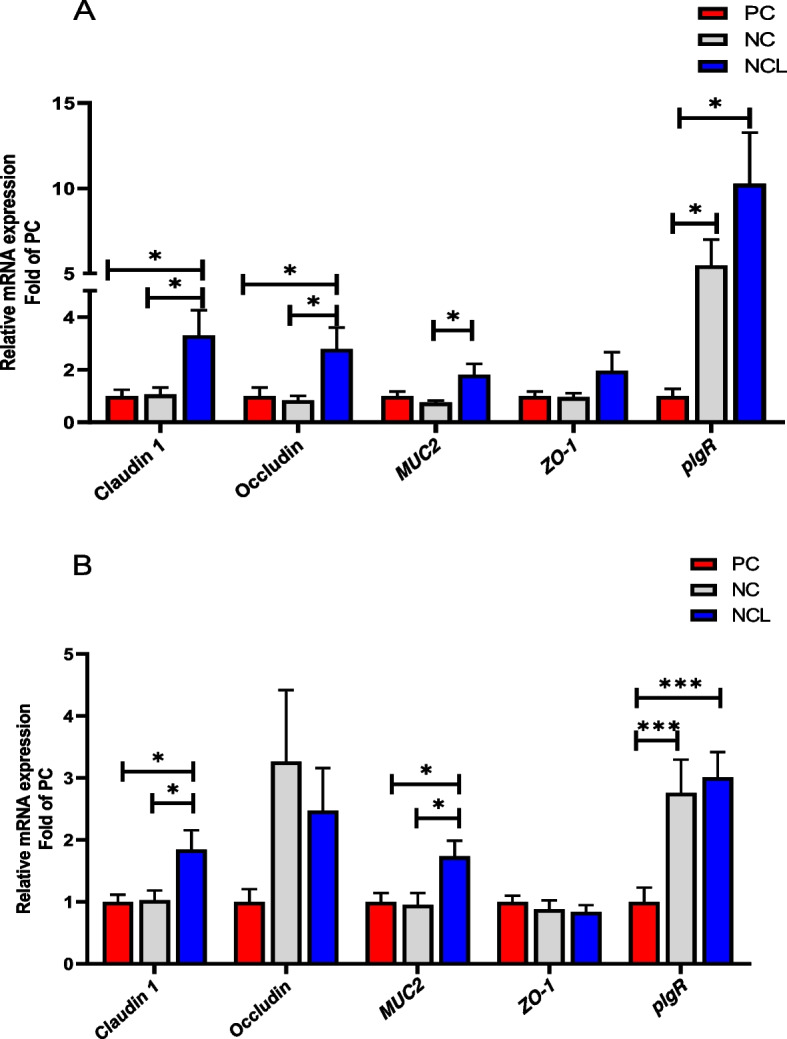
Gene expression of tight junction proteins in the intestine of broilers fed arginine deficient diets supplemented with arginine or *L-*citrulline. (**A**) Ileal mucosa (**B**) Jejunum tissue. PC: Positive control; NC: Negative control; NCL: Negative control + *L-*citrulline. Data are presented as mean ± SEM (*n* = 8). Data are significantly different at *P < 0.05; ***P < 0.001

The relative mRNA expression of tight junction proteins and immune markers in the ileal mucosa revealed that NCL diet significantly upregulated (*P* < 0.05) the expression of Claudin 1, Occludin, *MUC2,* and *pIgR* higher than the NC diet (Fig. 8A). Also, the Claudin 1 and Occludin expression were increased (*P* < 0.05) by NCL diet higher than the PC diet, whereas, NC diet upregulated *pIgR* expression higher than PC diet (Fig. 8A). The *ZO-1* expression was not affected by dietary treatments (*P* > 0.05).

In the jejunum, feeding with NCL diet upregulated the expression of Claudin 1*,* and *MUC2* higher than both the PC and NC diets (Fig. 8B). In addition, both NCL and NC diets significantly upregulated *pIgR* expression higher than the PC diet (*P* < 0.05). Similar to the ileal expression, *ZO-1* did not differ with dietary treatments (*P* > 0.05).

### Histomorphometric analysis of the intestine

Table 7 shows that the intestinal morphometry of broilers was significantly affected by dietary treatments, except in the duodenum, where the VH, CD, and VH/CD ratio were not changed (*P* > 0.05). In the jejunum, the VH was lowered by NC diet, whereas, it was increased with PC and NCL diets (*P* < 0.05). The jejunal CD was increased in the PC and NC groups but lowered by NCL diet (*P* < 0.05). Also, the VH/CD ratio of the jejunum was lowered by NC diet relative to the PC and NC diets (*P* < 0.05). The VH of the ileum was not changed by dietary treatment (*P* > 0.05), however, NC diet significantly increased the ileal CD compared to the PC and NCL diets, which were similar. In contrast, the VH/CD ratio was lowered by NC diet but increased in both the PC and NCL fed groups (*P* < 0.05).

**Table 7:** Intestinal morphometry of broilers fed arginine deficient diets supplemented with arginine or *L-*citrulline

Intestinal morphometry, µm		Dietary treatments			*P* value
		PC	NC	NCL	
Duodenum	VH	1650.63 ± 55.35	1559.87 ± 46.63	1707.39 ± 78.67	0.241
	CD	184.53 ± 13.14	197.86 ± 14.00	190.96 ± 9.90	0.753
	VH/CD	9.28 ± 0.58	8.20 ± 0.55	9.11 ± 0.54	0.350
Jejunum	VH	1239.9 ± 40.97^a^	959.67 ± 45.12^b^	1138.83 ± 40.04^a^	0.001
	CD	213.54 ± 10.12^a^	208.90 ± 5.12^a^	173.19 ± 9.31^b^	0.008
	VH/CD	5.88 ± 0.19^a^	4.60 ± 0.23^b^	6.76 ± 0.43^a^	0.001
Ileum	VH	719.14 ± 27.59	719.89 ± 21.84	700.93 ± 29.63	0.857
	CD	191.71 ± 4.61^b^	253.17 ± 10.34^a^	201.89 ± 13.10^b^	0.001
	VH/CD	3.76 ± 0.15^a^	2.87 ± 0.18^b^	3.56 ± 0.33^a^	0.034

*VH* Villus height, *CD* Crypt depth, *VH/CD* Villus height to Crypt depth ratio, *PC:* Positive control, *NC*: Negative control, *NCL:* Negative control + *L-*citrulline

Data are presented as mean ± SEM (*n* = 8). ^a,b^Means with different superscript letters are significantly different (*P* < 0.05) within the same row

## Discussion

The present study examined the effects of arginine or *L-*Cit supplementation to broilers under arginine-deficient conditions. It was found that although feeding with the arginine-deficient-NC diet did not elicit significant effects on the growth performance of broilers, it changed the free plasma AA profile, arginine metabolism, intestinal nutrient transport, and intestinal integrity of broilers. Corresponding with our study, Dao et al. [22] demonstrated that in laying hens fed with arginine deficient diet and supplemented with 0.35% arginine or 0.35% citrulline, the birds did not differ in their feed intake and egg production performance. According to the standard guidelines for arbor acre broiler management [23], under optimal management, environmental and nutritional conditions, a body weight of 978 g, along with cumulative feed intake of 1190 g is expected of broilers at 21 days of age. Relative to these standards, our study showed that the experimental birds had depreciated performance, such that at 21 days of age, broilers fed PC diet exhibited about 48% and 42% decline in BW and FI respectively, compared to the standard guidelines. This impaired performance of broilers may likely occur due to nutrient imbalance in the basal diet used in the study. The basal diets were formulated as arginine-deficient, and from the findings of the present study, it is evident that the NC diet could not support the optimal growth performance of broilers regardless of *L*-arginine or *L-*Cit supplementation. In line with these, several studies have also reported that feeding chickens with an arginine deficient diet impeded their growth responses and production performance [12, 24, 25]. Interestingly, both PC and NCL diets increased the relative weight of the thigh muscle, without obvious changes to the final BW of broilers. In line with this, it was reported that the absolute and relative weight of thigh muscles were linearly increased in broilers with increasing levels of digestible arginine/lysine ratio, probably due to the actions of arginine on muscle creatine synthesis [26]. Thus, the potential for increased muscle mass observed with arginine and *L-*Cit supplementation may be further investigated over longer periods since both AA are involved in protein turnover and protein synthesis [27, 28].

The ALT and AST enzymes are released into the bloodstream when the hepatic parenchymal cells are damaged, along with the associative effects of inflammation and oxidative stress [29]. In this study, PC diet increased the plasma ALT higher than NCL diet and further increased the AST levels higher than both NC and NCL diets. These may be suggestive of liver injury since these enzymes are key markers of liver dysfunction. Although arginine has been shown to exert beneficial effects on liver functioning [30, 31], it was also reported that arginine supplementation potentiated liver injury in mercury chloride-treated rats, which was characterized by elevated ALT and AST levels [32]. Coincidently, this may account for the increased ALT and AST levels with PC diet, such that the added arginine in PC fed broilers would have exerted burden on liver metabolism ultimately affecting its functions. Importantly, the NCL diet afforded protective effects by lowering both plasma ALT and AST compared to the PC diet. This corroborates the report that *L-*citrulline treatment decreased ALT, AST, and the ratio of ALT/AST in high-fat and cholesterol*-*fed rats [33]. In addition, feeding broilers with NC diet increased the plasma urate content higher than both the PC and NCL diets. This may be indicative of increased protein catabolism, exposure to stressors, and impaired hepato-renal functions [34, 35]. However, both arginine and *L-*Cit supplementation were able to reverse these effects by diminishing urate accumulation in the bloodstream.

An examination of the plasma AA profile showed that feeding broilers with NC diet increased the circulation of several essential and non-essential AA than the arginine fortified-PC diet. It is understood that inadequate arginine supply from the NC diet could have activated other metabolic routes for protein synthesis, leading to the increased availability of certain AA. This alteration in protein synthesis was also evidenced by the accumulation of urate, as well as the decrease in thigh muscle weight of NC-fed broilers. Under certain states such as stress and catabolic conditions, some non-essential AA may become essential, especially where the capacity of endogenously synthesized AA is exceeded [36]. In this study, it was found that broilers fed NC diet had increased circulating levels of several AA, except for arginine. We had hypothesized that *L-*Cit supplementation would promote arginine availability to meet the body’s metabolic needs since arginine is widely involved in several precursory roles including NO production [37]. Interestingly, NCL diet was able to augment the levels of arginine, citrulline, and ornithine in circulation, whereas, the PC diet could not support circulating citrulline levels. An important finding of this study was that the free plasma citrulline was decreased during arginine supplementation relative to arginine deficient condition. This supports the understanding that citrulline supply is increasingly effective during conditions of arginine deficiency. This also corroborates with previous reports where citrulline proved more effective in providing arginine than direct arginine supplementation [38–40]. Furthermore, it is necessary to report that the experimental birds were examined during the fed state, since the nutrient concentrations in body fluids are closely related to the food composition and anabolic responses of the body during the fed (postprandial) conditions [41, 42].

Since arginine is responsible for NO synthesis, we proposed that the increase in arginine availability would result in increased NO production, similar to previous reports [15, 37]. However, the results revealed that the plasma NO was unaffected by dietary treatments, whereas, *L-*Cit supplementation to NC diet promoted NO concentration higher than arginine supplementation in both the kidney and liver tissues. Thus, in line with our findings that NCL diet increased both the plasma arginine and citrulline content, it was evident that *L-*Cit could serve as a potential substrate to restore NO production where the arginine availability was limited. To corroborate this, dietary *L-*citrulline was reported to promote the circulating arginine and NO levels in a dose-dependent manner in laying hens [15]. In addition, it is established that citrulline is directly converted to argininosuccinate via the actions of ASS and further metabolized by ASL to produce arginine, which in turn can be converted to citrulline via NOS activity or ornithine via arginase actions [43–45]. Therefore, an investigation into these enzymes would provide succinct information on the metabolic transformations that occur during citrulline to arginine conversion in poultry. In the present study, PC diet stimulated higher ASS, and ASL activities than NCL diet, suggesting that supplemental arginine promoted citrulline conversion to arginine than direct *L-*Cit supplementation. Importantly, arginine serves as a substrate for three major NOS isoforms, including the constitutive forms of nNOS, which is found in the central nervous system, eNOS which is expressed in the vascular endothelium, and the inducible isoform, iNOS which is stimulated by inflammatory mediators, cytokines and stress signals [46, 47]. In this study, NCL diet induced plasma eNOS activity higher than the PC and NC diets, and also elevated the liver eNOS activity compared to the PC diet. It further upregulated the kidney *eNOS* expression higher than the NC diet. This may account for the increased liver NO concentration by NCL diet, that is, via eNOS catalysis of arginine to release NO. Contradictorily, PC diet was observed to induce kidney eNOS activity, the kidney expression of *nNOS* and *eNOS*, as well as *iNOS* expression in the liver. However, it exerted no stimulatory effect on NO production. These contrasting findings cannot be fully explained and may suggest differential responses between the kidney and liver during arginine metabolism in chickens.

Furthermore, earlier studies had demonstrated the absence of CPS 1 and OTC activity in chickens [48, 49]. In the present study, changes in the relative mRNA expression of *OTC* in the liver, as well as *OTC* and *CPS1* in the kidney were observed. This coincides with studies that had identified a functional *CPS1* gene in the brain, muscle, and immune tissues of chicken, although its role in the urea cycle was yet to be fully ascertained [50, 51]. In line with this, Li et al. [52] also reported an upregulated *CPS1* and *OTC* gene expression in the liver and breast muscle of chicken embryos following dietary *L-*arginine supplementation to broiler breeder hen’s diet. Noteworthy, the gene expression of *OTC* in the liver, as well as *OTC* and *CPS1* in the kidney were relatively higher than *nNOS* and *eNOS,* which were minimally expressed in the liver and kidney respectively. Hence, these findings suggest that both the kidney and liver are active sites for arginine metabolism in chickens, although with distinct peculiarities. Comparatively, it is reasonable that arginine to citrulline metabolism is more efficient in the kidney considering the relatively higher expression of *nNOS, ASS, ARG2,* and *OTC* in the kidney than in the liver. Concurrently, the kidney had been reported as the greatest site for citrulline to arginine conversion [53].

Arginase is an important enzyme that hydrolyzes arginine to produce ornithine and urea [54]. In chicken, the kidney arginase is important in regulating arginine metabolism, since arginine degradation is dependent on both the kidney arginase activity and the plasma arginine level [55]. Earlier works had reported on the molecular characterization of arginase enzyme in the kidney and liver tissues of chickens [56, 57]. Robbins and Baker [58] showed that arginine deficiency slightly increased the kidney arginase activity, however, increasing the arginine concentration in diets that had either 50% or 100% nitrogen requirement markedly increased the kidney arginase by twofold. Chu and Nesheim [55] also showed that arginine supplementation to laying hens induced higher arginase activity in the kidney than the low protein diet. Corresponding with these reports, the arginine-supplemented-PC diet promoted the plasma, kidney, and liver arginase 1 activity higher than *L-*Cit supplemented-NCL diets. Interestingly, citrulline can exert a non-competitive inhibition on arginase catalysis of arginine to attenuate arginase actions [59]. In this study, NCL diet persistently diminished the arginase 1 activity in the plasma, kidney, and liver. This was also corroborated by the downregulated *ARG2* gene expression in the liver, but not in the kidney. Moreover, *L-*Cit inhibition of kidney and liver arginase 1 activity corresponded with its induction of NO concentration in these tissues. Furthermore, arginase directly competes with NOS for the substrate, arginine [60]. Thus, it was also found that *L-*Cit inhibition of arginase may have led to its induction of eNOS activity probably to augment NO production.

Studies have shown that arginine is crucial to intestinal health since it maintains intestinal integrity, improves gut immunity, intestinal absorption, gut barrier functions and protects the gut microbial composition [9, 10]. Importantly, different AA transporter systems in the brush border membranes are responsible for the transport of specific free AA into the enterocytes [61]. The b^0+^AT is a light chain homolog of the heteromeric AA transporter which functions to maintain homeostasis of AA pools in various tissues [62]. EAAT3 is an important anionic AA transporter with an affinity for glutamate [63]. Also, PepT1 is an AA transport system that facilitates the absorption of dietary AA as dipeptides and tripeptides into the intestinal epithelial cells [64]. The present study showed that NCL diet upregulated *PepT1* expression in the ileum and jejunum tissues, compared to the PC and NC diet or PC diet, respectively. It was reported that the expression of chicken *PepT1* gene was regulated in response to changes in the dietary crude protein and AA levels [65, 66]. In another study, *PepT1* expression was modulated in malnourished rats despite atrophic changes in the intestinal mucosa [67]. This may explain the increased *PepT1* expression in the jejunum of NC-fed broilers. Furthermore, citrulline can be transported efficiently across the intestinal lumen by Na^+^-dependent (system B^0,+^) and Na^+^-independent (systems L and b^0,+^) transporters [16]. Similarly, the cellular transport of arginine involves transit via the system y^+^ and Na-dependent transporters (e.g., b^0,+^, B^0,+^, and y^+^L) in a cell*-*specific manner [68]. Thus, the upregulation of *PepT1* and *EAAT3* systems with NCL diet suggests that *L-*Cit supplementation elicited a positive influence on intestinal AA transport with profound effects on AA uptake in broilers.

Tight junction proteins such as Claudin, Occludin, and ZO-1 play important roles in the intestinal epithelial barrier, and as such, they are crucial for nutrient absorption and intestinal immunity [11, 69]. In addition, pIgR is used to establish the first lines of intestinal defense since it transports the polymeric Immunoglobulin A to the intestinal lumen and across epithelial cells [70]. In this study, NCL diet enhanced Claudin 1 and Occludin expression in the ileal mucosa greater than the PC and NC diets. Alongside this, the jejunal Claudin 1 and *MUC2* expression were upregulated by NCL diet compared to the PC and NC diets, while the *pIgR* expression was also increased by both NCL and NC diets relative to PC diet. Previous studies have shown that arginine is necessary for the maintenance of intestinal epithelial integrity [71], while citrulline is an important biomarker of gut health and intestinal functions [44, 72, 73]. Therefore, these findings suggest that *L-*Cit supplementation enhanced the expression of intestinal tight junction proteins, and promoted intestinal mucosal defenses better than arginine supplementation during arginine deficiency. Thus, citrulline may serve as a functional AA during conditions of low arginine availability to support arginine -mediated roles such as nutrient absorption and intestinal immunity.

Proper maintenance of the intestinal morphology is beneficial for nutrient digestion, absorption, and optimal broiler performance. In this study, the VH/CD ratio was lowered in the ileum, while the VH and VH/CD ratios were decreased in the jejunum of NC-fed broilers compared to the PC and NCL groups. This suggests that the nutrient absorption area and efficiency of absorption were compromised in NC-fed broilers, however, supplementation with either arginine or *L-*Cit would alleviate the gut barrier failure. To corroborate this, studies have shown that *L-*arginine supplementation improved VH and preserved the jejunal morphology in *Clostridium perfringens* infected broilers [10], as well as the VH and VH/CD ratio in coccidiosis-challenged chickens [74]. The beneficial effects of arginine on intestinal microstructure and morphometry have been attributed to its role in the synthesis of polyamines and NO, since these metabolites are vital for intestinal development and nutrient absorption [75]. In addition, citrulline concentration is significantly correlated with intestinal markers of crypt depth, and VH/CD ratio [76], such that plasma citrulline concentration is decreased during conditions of intestinal villus atrophy [77]. Therefore, this study demonstrates that arginine and *L-*Cit were beneficial in promoting intestinal nutrient absorption, barrier integrity, and gut health during arginine -deficient conditions.

## Conclusion

**Fig. 9:**
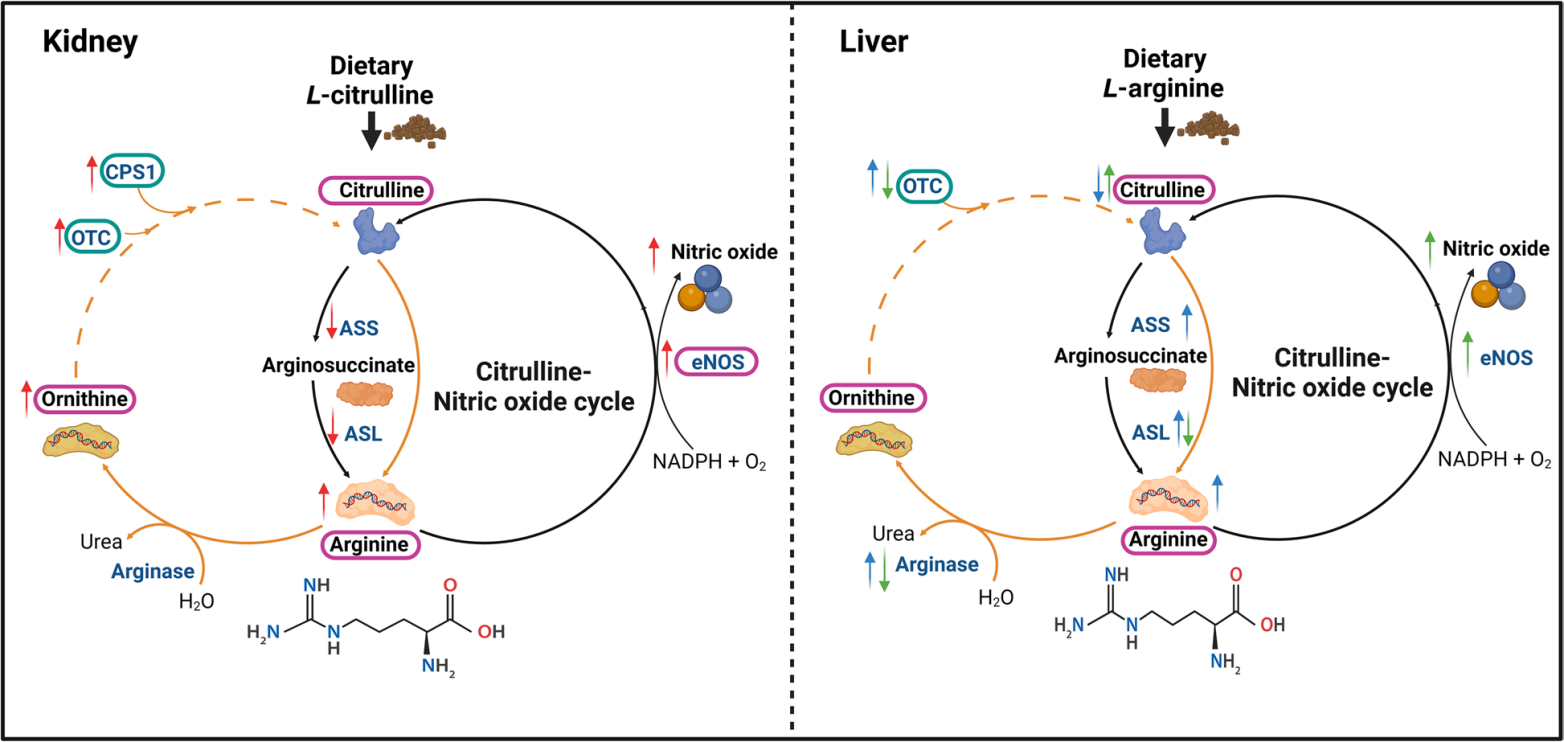
Schematic representation of Citrulline–Nitric oxide cycle depicting arginine to citrulline conversion in the kidney and liver tissues. From this study, it was demonstrated that dietary *L-*Cit supplementation to arginine deficient broilers would promote the circulating arginine and ornithine levels, as well as eNOS activity. With the kidney as the main site for citrulline metabolism, *L-*Cit supplementation during arginine deficiency diminished ASS/ASL activity, whereas, it upregulated *OTC* and *CPS1* genes. Furthermore, it induced NO production via eNOS catalysis of arginine. In the liver, arginine supplementation during arginine deficiency promoted the circulating arginine levels and induced ASS/ASL activity but it could not support citrulline supply. This coincided with the increased arginase activity and upregulated OTC expression. However, comparison between *L-*Cit and arginine supplementation during arginine deficiency revealed that *L*-Cit could sustain citrulline supply, inhibit arginase, and promote eNOS synthesis of NO in the liver greater than direct arginine supplementation. Importantly, uricotelic species including chickens are reportedly lacking the CPS1 and OTC enzyme for citrulline’s synthesis from ornithine, thus this was depicted with dotted lines. Legend: Upward arrow indicates an increase, while downward arrow indicates a decrease. Red arrows indicates the effects of dietary *L-*citrulline supplementation to arginine deficient broilers. Blue arrows indicates the effects of dietary *L-*arginine supplementation to arginine deficient broilers. Green arrows indicates the comparison between *L-*citrulline and *L-*arginine supplementation to arginine deficient broilers.  indicates plasma concentration.  indicates relative mRNA expression in target tissues

Taken together, *L-*Cit supplementation effectively replaced arginine in broiler’s diet owing to the ability of *L-*Cit to augment circulating arginine and subsequently tissue NO production (Fig. 9). Therefore, *L-*Cit provided as a crystalline AA to broilers would present an immediate metabolic precursor for arginine supply, tissue NO generation, facilitate nutrient transport and support intestinal integrity under conditions of arginine deficiency. Further studies expounding on the arginine-sparing effects of *L-*Cit would prove useful in optimizing dietary *L-*Cit in poultry feeding programs.

## Data Availability

All relevant data are provided within the paper and the dataset used in this study is fully available from the corresponding author.

## Abbreviations

AA: Amino acids
ALT: Alanine aminotransferase
ASS: Argininosuccinate synthetase
ASL: Argininosuccinate lyase
ARG2: Arginase 2
AST: Aspartate aminotransferase
*b*^0,+^AT: B^0,+^ AA transporter
BW: Bodyweight
BWG: Bodyweight gain
CD: Crypt depth
CK: Creatine kinase
CPS1: Carbamoyl phosphate synthetase 1
EAAT3: Excitatory amino acid transporter 3
eNOS: Endothelial nitric oxide synthase
FI: Feed intake
FCR: Feed conversion ratio
GLU: Glucose
iNOS: Inducible nitric oxide synthase
*L*-Cit: *L*-Citrulline
LDH: Lactate dehydrogenase
MUC2: Mucin 2
NO: Nitric oxide
nNOS: Neuronal nitric oxide synthase
OTC: Ornithine transcarbamylase
PepT1: Peptide transporter 1
pIgR: Polymeric Ig receptor
TG: Triglyceride
TCHO: Total cholesterol
tNOS: Total nitric oxide synthase
VH: Villus height
VH/CD: Villus height to Crypt depth ratio
ZO-1: Zona occludens 1

## References

[1] DeGroot AA, Braun U, Dilger RN (2019). Guanidinoacetic acid is efficacious in improving growth performance and muscle energy homeostasis in broiler chicks fed arginine-deficient or arginine-adequate diets. Poult Sci.

[2] Teng PY, Choi J, Yadav S, Tompkins YH, Kim WK (2021). Effects of low-crude protein diets supplemented with arginine, glutamine, threonine, and methionine on regulating nutrient absorption, intestinal health, and growth performance of eimeria-infected chickens. Poult Sci.

[3] Liu SY, Macelline SP, Chrystal PV, Selle PH (2021). Progress towards reduced-crude protein diets for broiler chickens and sustainable chicken-meat production. J Anim Sci Biotechnol.

[4] Belloir P, Méda B, Lambert W, Corrent E, Juin H, Lessire M (2017). Reducing the cp content in broiler feeds: Impact on animal performance, meat quality and nitrogen utilization. Animal.

[5] Chrystal PV, Greenhalgh S, Selle PH, Liu SY (2020). Facilitating the acceptance of tangibly reduced-crude protein diets for chicken-meat production. Anim Nutr.

[6] Castro FLS, Teng P, Yadav S, Gould RL, Craig S, Pazdro R (2020). The effects of l-arginine supplementation on growth performance and intestinal health of broiler chickens challenged with eimeria spp. Poult Sci.

[7] Wu G (2010). Functional amino acids in growth, reproduction, and health. Adv Nutr.

[8] Wu G (2013). Functional amino acids in nutrition and health. Amino Acids.

[9] Zhang B, Li G, Shahid MS, Gan L, Fan H, Lv Z (2020). Dietary l-arginine supplementation ameliorates inflammatory response and alters gut microbiota composition in broiler chickens infected with salmonella enterica serovar typhimurium. Poult Sci.

[10] Zhang B, Lv Z, Li Z, Wang W, Li G, Guo Y (2018). Dietary l-arginine supplementation alleviates the intestinal injury and modulates the gut microbiota in broiler chickens challenged by clostridium perfringens. Front Microbiol.

[11] Kidd MT, Maynard CW, Mullenix GJ (2021). Progress of amino acid nutrition for diet protein reduction in poultry. J Anim Sci Biotechnol.

[12] Wang C, Zheng AJ, Xie M, Huang W, Xie JJ, Hou SS (2014). Hypothalamic protein profiles associated with inhibited feed intake of ducks fed with insufficient dietary arginine. Animal.

[13] Khalaf D, Krüger M, Wehland M, Infanger M, Grimm D (2019). The effects of oral l-arginine and l-citrulline supplementation on blood pressure. Nutrients.

[14] Dao HT, Sharma NK, Bradbury EJ, Swick RA (2021). Response of meat chickens to different sources of arginine in low-protein diets. J Anim Physiol Anim Nutr.

[15] Uyanga VA, Jiao H, Zhao J, Wang X, Lin H (2020). Dietary l-citrulline supplementation modulates nitric oxide synthesis and anti-oxidant status of laying hens during summer season. J Anim Sci Biotechnol.

[16] Bahri S, Zerrouk N, Aussel C, Moinard C, Crenn P, Curis E (2013). Citrulline: From metabolism to therapeutic use. Nutrition.

[17] Edmonds M, Lowry K, Baker D (1987). Urea cycle metabolism: Effects of supplemental ornithine or citrulline on performance, tissue amino acid concentrations and enzymatic activity in young pigs fed arginine-deficient diets. J Anim Sci.

[18] NRC. Nutrient requirements of poultry. 9th revised edition. Washington: National Academy Press; 1994.

[19] Chen J, Tellez G, Richards JD, Escobar J (2015). Identification of potential biomarkers for gut barrier failure in broiler chickens. Front Vet Sci.

[20] AOAC International. Official methods of analysis in association of official analytical chemists.18th ed. Gaithersburg, MD:AOAC International; 2006.

[21] Sun M, Ma N, Liu H, Liu Y, Zhou Y, Zhao J (2022). The optimal dietary arginine level of laying hens fed with low-protein diets. J Anim Sci Biotechnol.

[22] Dao HT, Sharma NK, Bradbury EJ, Swick RA (2021). Response of laying hens to *L*-arginine, *L*-citrulline and guanidinoacetic acid supplementation in reduced protein diet. Anim Nutr.

[23] Aviagen. Arbor Acre broiler nutrition specifications. Aviagen Incorporated, USA. 2019;0419-AVNAA-043. http://tmea.aviagen.com/assets/Tech_Center/AA_Broiler/AABroilerNutritionSpecs2019-EN.pdf. Accessed 24 Feb 2022.

[24] DeGroot AA, Braun U, Dilger RN (2018). Efficacy of guanidinoacetic acid on growth and muscle energy metabolism in broiler chicks receiving arginine-deficient diets. Poult Sci.

[25] Xu YQ, Guo YW, Shi BL, Yan SM, Guo XY (2018). Dietary arginine supplementation enhances the growth performance and immune status of broiler chickens. Livestock Sci.

[26] Corzo A, Lee J, Vargas JI, Silva M, Pacheco WJ (2021). Determination of the optimal digestible arginine to lysine ratio in Ross 708 male broilers. J Appl Poult Res.

[27] Wang R, Jiao H, Zhao J, Wang X, Lin H (2018). L-arginine enhances protein synthesis by phosphorylating mTOR (Thr 2446) in a nitric oxide-dependent manner in C2C12 cells. Oxid Med Cell Longev.

[28] Breuillard C, Goron A, Moinard C, Walrand S (2019). Chapter 20 - Citrulline and skeletal muscle. Nutrition and skeletal muscle.

[29] Mokhtar, Fathi M, Kambiz N, Adl, Nezhad E, Habib A, et al. The role of oxidative stress in development of congestive heart failure (CHF) in broiler with pulmonary hypertension syndrome (PHS). J Anim Vet Adv. 2011;10:724–29.

[30] Li Q, Liu Y, Che Z, Zhu H, Meng G, Hou Y (2012). Dietary l-arginine supplementation alleviates liver injury caused by *Escherichia coli* LPS in weaned pigs. Innate Immun.

[31] Ozsoy Y, Coskun T, Yavuz K, Ozbilgin K, Var A, Ozyurt B (2011). The effects of *L*-arginine on liver damage in experimental acute cholestasis an immunohistochemical study. HPB Surg.

[32] Nikolic J, Stojanovic I, Pavlovic R, Sokolovic D, Bjelakovic G, Beninati S (2007). The role of *L*-arginine in toxic liver failure: Interrelation of arginase, polyamine catabolic enzymes and nitric oxide synthase. Amino Acids.

[33] El-Kirsh AAA, Abd El-Wahab HMF, Abd-Ellah Sayed HF (2011). The effect of *L*-arginine or *L* -citrulline supplementation on biochemical parameters and the vascular aortic wall in high-fat and high-cholesterol-fed rats. Cell Biochem.

[34] Saka WA, Akhigbe RE, Abidoye AO, Dare OS, Adekunle AO (2021). Suppression of uric acid generation and blockade of glutathione dysregulation by*L*-arginine ameliorates dichlorvos-induced oxidative hepatorenal damage in rats. Biomed Pharmacother.

[35] Qaid MM, Al-Garadi MA. Protein and amino acid metabolism in poultry during and after heat stress: A review. Animals (Basel). 2021;11(4):1167. 10.3390/ani11041167 PMC8074156 33921616

[36] Morris CR, Hamilton-Reeves J, Martindale RG, Sarav M, Ochoa Gautier JB (2017). Acquired amino acid deficiencies: A focus on arginine and glutamine. Nutr Clin Pract.

[37] Ale Saheb Fosoul SS, Azarfar A, Gheisari A, Khosravinia H (2019). Performance and physiological responses of broiler chickens to supplemental guanidinoacetic acid in arginine-deficient diets. British Poult Sci.

[38] Schwedhelm E, Maas R, Freese R, Jung D, Lukacs Z, Jambrecina A (2008). Pharmacokinetic and pharmacodynamic properties of oral *L*-citrulline and *L*-arginine: Impact on nitric oxide metabolism. Br J Clin Pharmacol.

[39] Agarwal U, Didelija IC, Yuan Y, Wang X, Marini JC (2017). Supplemental citrulline is more efficient than arginine in increasing systemic arginine availability in mice. J Nutr.

[40] Wijnands KA, Vink H, Briede JJ, van Faassen EE, Lamers WH, Buurman WA (2012). Citrulline a more suitable substrate than arginine to restore no production and the microcirculation during endotoxemia. PLoS ONE.

[41] Holecek M, Sispera L (2016). Effects of arginine supplementation on amino acid profiles in blood and tissues in fed and overnight-fasted rats. Nutrients.

[42] Everaert N, Decuypere E, Buyse J. Feed intake and regulation. In: Hendriks WH, Verstegen MWA, Babinszky L, editors. Poultry and pig nutrition: Challenges of the 21st century. Wageningen: Academic Publishers; 2019. p. 59–75.

[43] Ouchi Y, Chowdhury VS, Cockrem JF, Bungo T (2021). Effects of thermal conditioning on changes in hepatic and muscular tissue associated with reduced heat production and body temperature in young chickens. Front Vet Sci.

[44] Uyanga VA, Amevor FK, Liu M, Cui Z, Zhao X, Lin H (2021). Potential implications of citrulline and quercetin on gut functioning of monogastric animals and humans: A comprehensive review. Nutrients.

[45] Cynober L, Moinard C, De Bandt JP (2010). The 2009 ESPEN Sir David Cuthbertson. Citrulline: A new major signaling molecule or just another player in the pharmaconutrition game?. Clin Nutr.

[46] Allerton TD, Proctor DN, Stephens JM, Dugas TR, Spielmann G, Irving BA (2018). L-citrulline supplementation: Impact on cardiometabolic health. Nutrients.

[47] Sorokin A (2016). Nitric oxide synthase and cyclooxygenase pathways: A complex interplay in cellular signaling. Curr Med Chem.

[48] Tamir H, Ratner S (1963). Enzymes of arginine metabolism in chicks. Arch Biochem Biophys.

[49] Wu G, Flynn NE, Yan W, Barstow DG, Jr. Glutamine metabolism in chick enterocytes: Absence of pyrroline-5-carboxylase synthase and citrulline synthesis. Biochem J. 1995;306 ( Pt 3)(Pt 3):717–21. 10.1042/bj3060717 PMC1136580 7702565

[50] International Chicken Genome Sequencing Consortium (2004). Sequence and comparative analysis of the chicken genome provide unique perspectives on vertebrate evolution. Nature.

[51] Shimogiri T, Bosak N, Morisson M, Okamoto S, Kawabe K, Maeda Y (2004). Assignment of CPS1, OTC, CRYD2, ARG2 and ASS genes to the chicken Rh map. Genetics, selection, evolution: GSE.

[52] Li F, Ning H, Duan X, Chen Z, Xu L (2021). Effect of dietary *L*-arginine of broiler breeder hens on embryonic development, apparent metabolism, and immunity of offspring. Domest Anim Endocrinol.

[53] Fernandes J, Murakami A. Arginine metabolism in uricotelic species. Acta Sci Anim Sci. 2010;32(4):357–66.

[54] Wu G, Bazer FW, Davis TA, Kim SW, Li P, Marc Rhoads J (2009). Arginine metabolism and nutrition in growth, health and disease. Amino Acids.

[55] Chu SW, Nesheim MC (1979). The relationship of plasma arginine and kidney arginase activity to arginine degradation in chickens. J Nutr.

[56] Grazi E, Magri E (1972). Molecular characteristics of chicken liver arginase. Biochem J.

[57] Traniello S, Barsacchi R, Magri E, Grazi E (1975). Molecular characteristics of chicken kidney arginase. Biochem J.

[58] Robbins KR, Baker DH (1981). Kidney arginase activity in chicks fed diets containing deficient or excessive concentrations of lysine, arginine, histidine, or total nitrogen. Poult Sci.

[59] Hunter A, Downs CE (1945). The inhibition of arginase by amino acids. J Biol Chem.

[60] El-Bassossy HM, El-Fawal R, Fahmy A, Watson ML (2013). Arginase inhibition alleviates hypertension in the metabolic syndrome. Br J Pharmacol.

[61] Wang J, Lin J, Wang J, Wu S, Qi G, Zhang H (2020). Effects of in ovo feeding of n-acetyl-l-glutamate on early intestinal development and growth performance in broiler chickens. Poult Sci.

[62] Wu D, Grund TN, Welsch S, Mills DJ, Michel M, Safarian S (2020). Structural basis for amino acid exchange by a human heteromeric amino acid transporter. Proc Natl Acad Sci U S A.

[63] Hundal HS, Taylor PM (2009). Amino acid transceptors: Gate keepers of nutrient exchange and regulators of nutrient signaling. Am J Physiol Endocrinol Metab.

[64] Nässl AM, Rubio-Aliaga I, Sailer M, Daniel H (2011). The intestinal peptide transporter PEPT1 is involved in food intake regulation in mice fed a high-protein diet. PLoS ONE.

[65] Frazier S, Ajiboye K, Olds A, Wyatt T, Luetkemeier E, Wong E (2008). Functional characterization of the chicken peptide transporter 1 (PEPT1, SLC15A1) gene. Anim Biotechnol.

[66] Shiraga T, Miyamoto K, Tanaka H, Yamamoto H, Taketani Y, Morita K (1999). Cellular and molecular mechanisms of dietary regulation on rat intestinal h+/peptide transporter PEPT1. Gastroenterology.

[67] Ihara T, Tsujikawa T, Fujiyama Y, Bamba T (2000). Regulation of PEPT1 peptide transporter expression in the rat small intestine under malnourished conditions. Digestion.

[68] Grillo MA, Lanza A, Colombatto S (2008). Transport of amino acids through the placenta and their role. Amino Acids.

[69] Zhao X, Wang L, Zhu C, Xia X, Zhang S, Wang Y, et al. The antimicrobial peptide mastoparan x protects against enterohemorrhagic *Escherichia coli* o157:H7 infection, inhibits inflammation, and enhances the intestinal epithelial barrier. Front Microbiol. 2021;12:644887. 10.3389/fmicb.2021.644887 PMC8222680 34177825

[70] Zhang Q, Eicher SD, Applegate TJ (2015). Development of intestinal mucin 2, IgA, and polymeric Ig receptor expressions in broiler chickens and pekin ducks. Poult Sci.

[71] Xia M, Ye L, Hou Q, Yu Q (2016). Effects of arginine on intestinal epithelial cell integrity and nutrient uptake. British J Nutr.

[72] Batista MA, Nicoli JR, dos Santos MF, Nogueira Machado JA, Esteves Arantes RM, Pacífico Quirino IE (2012). Pretreatment with citrulline improves gut barrier after intestinal obstruction in mice. J Parenteral Enteral Nutr.

[73] Papadia C, Dhaliwal W, Kelly P, Corazza GR, Franzè A, Di Sabatino A (2009). Plasma citrulline as a quantitative biomarker of HIV-associated duodenal mucosal damage in a tropical enteropathy population. Dig Liv Dis.

[74] Yazdanabadi FI, Mohebalian H, Moghaddam G, Abbasabadi M, Sarir H, Vashan SJH (2020). Influence of *Eimeria* spp. Infection and dietary inclusion of arginine on intestine histological parameters, serum amino acid profile and ileal amino acids digestibility in broiler chicks. Vet Parasitol.

[75] Abdulkarimi R, Shahir MH, Daneshyar M (2019). Effects of dietary glutamine and arginine supplementation on performance, intestinal morphology and ascites mortality in broiler chickens reared under cold environment. Asian-Australas J Anim Sci.

[76] Papadia C, Kelly P, Caini S, Roberto Corazza G, Shawa T, Franzè A (2010). Plasma citrulline as a quantitative biomarker of HIV-associated villous atrophy in a tropical enteropathy population. Clin Nutr.

[77] Crenn P, Vahedi K, Lavergne-Slove A, Cynober L, Matuchansky C, Messing B (2003). Plasma citrulline: A marker of enterocyte mass in villous atrophy-associated small bowel disease. Gastroenterology.

